# Representation and the Voluntary System

**Published:** 1913-09-27

**Authors:** James Henry Smethurst

**Affiliations:** Chairman of the Warrington Infirmary.


### THE EDITOR'S LETTER-BOX.

### "Representation and the Voluntary System."

*To the Editor of* The Hospital.

Sir,—In your issue of the 13th inst. appeared a most interesting account of the progress made in remodelling the North Staffordshire Infirmary, and the success of this must have been extremely gratifying not only to the president and members of his committee, but also to the medical superintendent, Dr. T. Basil Rhodes, who is recognised as a specialist in the art of raising money and using it to the best advantage.

In order to benefit other hospital authorities, Dr. Rhodes issued a pamphlet giving his own experiences, and his ideas as to representation on the management committee have in my opinion been so thoroughly misunderstood by one of your correspondents that there appears in the same issue of your paper an article which compels me to reply and, as far as I can, support the theory of Dr. Rhodes, who states most clearly in effect that he wished those workers who are paying money year after year to the general funds of the infirmary to send representatives to see how that money is spent and at the same time help the rest of the committee to understand the point of view of the sick people and their friends.

This is a far different thing than telling the workpeople that if they subscribe so much money, then they shall have so much representation. The writer of the criticism has evidently had no experience of the type of representative that has evidently been sent to the North Staffordshire Infirmary, or to that institution over which I have the honour to preside.

From my own experience no keener advocate for the hospital funds can be found, and no sterner critic of those who try to abuse the benefits provided, than the representatives I have come in contact with. The day of "sleepy" committees making spasmodic appeals has gone.

Our hospitals are increasingly becoming an absolute necessity, and funds should be provided in the most dignified and businesslike ways possible, and one of these methods is the arranging for systematic contributions by those who are unfortunately compelled on many occasions to take advantage of their resources; and I personally should be glad to give the writer of the criticism full particulars of what has been done here if he cares to communicate with me.—Yours faithfully,

James Henry Smethurst,

Chairman of the Warrington Infirmary.

September 23, 1913.

[If Mr. Smethurst will kindly forward us the particulars to which he refers we shall have pleasure in considering them for publication.—Ed. The Hospital.]

